# Temporal evolution of primary angiitis of the central nervous system (PACNS) on MRI following immunosuppressant treatment

**DOI:** 10.1186/s13244-024-01710-y

**Published:** 2024-06-09

**Authors:** Franca Wagner, Gonçalo G. Almeida, Erik P. Willems, Johannes Weber, Johannes Geiss, Thomas Hundsberger, Pasquale Mordasini, Simon Wildermuth, Sebastian Leschka, Stephan Waelti, Tobias Johannes Dietrich, Tim Steffen Fischer

**Affiliations:** 1 Bern University Hospital, University of Bern, Bern, Switzerland; 2 University of Bern, Bern, Switzerland; 3 Division of Radiology and Nuclear Medicine, Cantonal Hospital St. Gallen, St. Gallen, Switzerland; 4 Clinical Trials Unit, Cantonal Hospital St. Gallen, St. Gallen, Switzerland; 5 Division of Radiology and Nuclear Medicine, Cantonal Hospital St. Gallen, Medical School St. Gallen, St. Gallen, Switzerland; 6 Department of Neurology and Oncology, Cantonal Hospital St. Gallen, Medical School St. Gallen, St. Gallen, Switzerland

**Keywords:** Magnetic resonance imaging, Brain, Vasculitis, Contrast media

## Abstract

**Purpose:**

To systematically analyse the time course of vessel wall enhancement and associated stenosis in patients with primary angiitis of the central nervous system (PACNS) following immunosuppressive therapy.

**Material and methods:**

Two neuroradiologists retrospectively analysed MRIs of patients with PACNS seen at the Bern University Hospital and the St. Gallen Cantonal Hospital between 2015 and 2020. MRIs were examined for the presence of vessel wall enhancement, length of vessel wall enhancement (mm), circumferential extent of enhancement (degree) and degree of stenosis (%). Descriptive statistics and measurements of interobserver reliability were obtained. To investigate the temporal profiles of the variables following the commencement of immunosuppressant treatment, four series of Bayesian generalised multi-level models were generated.

**Results:**

A total of 23 patients with 43 affected vessels identified from 209 MRI exams were evaluated (mean follow-up: 715 days, standard deviation ± 487 days), leading to a complete dataset of 402 entries. Vessel wall enhancement and circumferential extent of enhancement decreased for approximately 1 year after the initiation of immunosuppressant therapy. Changes were more pronounced in younger patients. Disappearance of vessel wall enhancement (in at least one vessel) was seen in about half of patients after a median of 172 days interquartile range 113–244, minimum 54 days, maximum 627 days.

**Conclusions:**

This study evaluated the typical time course of vessel wall enhancement in patients with PACNS. Our results could be a useful reference for radiologists and clinicians interpreting follow-up imaging in patients with PACNS.

**Critical relevance statement:**

Routine clinical exams can be interpreted with more confidence when radiologists are aware of the typical temporal evolution of vessel wall enhancement in patients with primary angiitis of the central nervous system after initiation of immunosuppressive therapy.

**Key Points:**

- Few data exist for vessel wall imaging of primary angiitis of the central nervous system.
- Following immunosuppressant therapy, vessel wall enhancement decreases for approximately one year.
- These results may serve as a reference for radiologists performing follow-up imaging.

**Graphical Abstract:**

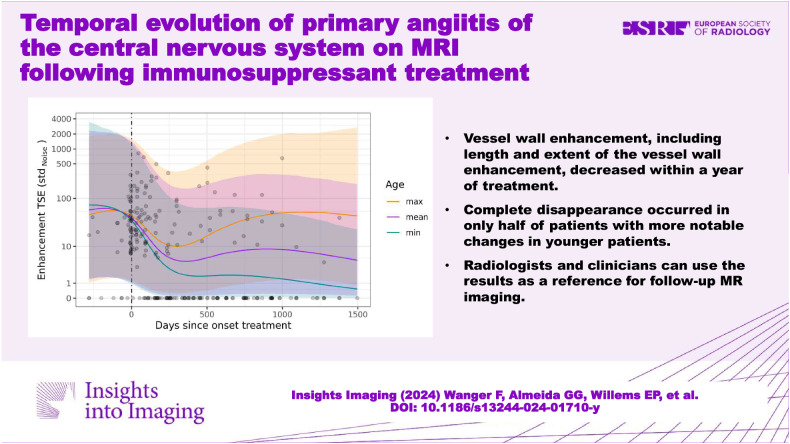

## Introduction

Primary angiitis of the central nervous system (PACNS) is a rare and severe form of autoimmune disease that is limited to the brain, spinal cord, and leptomeninges [1, 2]. The estimated prevalence in North America is 2.4/1,000,000 person-years [3]. Ischaemia, and less frequently haemorrhage, are typical manifestations [4]. Microvascular and macrovascular disease and reversible cerebral vasoconstriction syndrome, in particular are important differential diagnoses on imaging [5]. Symptoms include headache, altered cognition, and focal neurological deficits [1, 2]. Due to the nonspecific symptoms they cannot be used to discriminate between PACNS and other diseases [6]. The disease affects all age groups but an increase in incidence is observed with older age [1, 3]. Histopathologically, PACNS is characterised by granulomatous inflammation, lymphocytic cellular infiltrates and necrotising vasculitis [4].

Brain biopsy is important for the histological diagnosis of PACNS but is associated with false positive or false negative findings [7] and has a sensitivity between 53 and 63% [8]. Furthermore, the likelihood of sampling errors from areas of non-affected brain regions has to be weighed against the risk of biopsy in eloquent brain regions.

PACNS is considered to be likely when confirmation by biopsy was not attempted or was not possible, but the diagnosis was established based on digital subtraction angiography (DSA) and MRI findings in addition to a characteristic cerebrospinal fluid profile (CSF) and after exclusion of mimics [2]. DSA may also be included in the diagnostic workup of PACNS, but findings are unremarkable in small-vessel vasculitis and nonspecific in medium-sized vessel vasculitis [2]. Hence, the overall sensitivity has been reported as 40–90%, with a very low specificity of only 30% [9]. CSF analyses are abnormal in 66–90% of patients with CNS vasculitis showing lymphomonocytic pleocytosis or elevated protein levels [2, 6, 10].

Conventional MRI sequences are usually abnormal in patients with PACNS, but findings are nonspecific and most commonly include infarction or haemorrhagic lesions of different ages [11]. Dedicated vessel wall imaging with an application of techniques to suppress the intraluminal blood signal “black blood” or “dark blood” is therefore currently used to detect inflammatory changes within the vessel wall [5, 12, 13]. To the best of our knowledge, few studies have evaluated vessel wall imaging in patients with PACNS [14–17].

The aim of this study was to evaluate the temporal evolution of vessel inflammation following immunosuppressant treatment in patients with PACNS.

## Material and methods

### Patient selection

This study received Institutional Review Board (Ethics Committee of Eastern Switzerland) approval and written informed consent was obtained from all patients. A retrospective search of the databases of the University Hospital Bern and the St. Gallen Cantonal Hospital, Switzerland, was carried out to identify patients diagnosed with PACNS between 2015 and 2020. The inclusion criteria were: all patients had to be diagnosed by the Department of Neurology following an interdisciplinary discussion by the neurovascular board, besides imaging including vessel wall imaging, a lumbar puncture was required. Biopsy and digital subtraction angiography were performed based on the board’s recommendation in less clear cases. Moreover, immunosuppressant therapy had to be administered for at least 6 weeks; a short course of high-dose corticosteroids only did not qualify as immunosuppressant therapy in this study. Baseline MRI was required prior to the start of the immunosuppressant therapy up to one year before the first onset of CNS vasculitis-related symptoms, including a diffusion-weighted image, intracranial 3D-time of flight (TOF) MR angiography and a T1-weighted post-contrast “dark blood” sequence for evaluation of vessel wall enhancement.

### Imaging

Imaging was performed at the Institute of Diagnostic and Interventional Neuroradiology of the University Hospital Bern or at the Department of Radiology and Nuclear Medicine of the St. Gallen Cantonal Hospital. Due to the multicentric nature of this study, MRI scanners and imaging protocols varied. Most scanners were provided by Siemens Healthineers, Erlangen, Germany, few scanners were provided by Philips Health Systems, Gland, Switzerland. MRI protocols consisted of diffusion-weighted imaging with a 0 and 1000 *b*-value for calculation of the apparent diffusion coefficient and an axial T2-weighted sequence with or without fluid attenuation for CSF suppression (e.g., fluid attenuated inversion recovery FLAIR). Intracranial MR angiography was performed without contrast using 3D-TOF. A T1-weighted, blood suppressed “dark blood” sequence was used to evaluate vessel wall enhancement, either as turbo spin echo (TSE) or sampling perfection with application of optimised contrast using different flip angle evolution (SPACE). Vessel wall imaging (dark blood sequences) was acquired in 3-T scanners only. At both study centres the post-contrast scans were performed after 1 min of contrast injection (0.1 mmol/kilogram body weight; Gadovist, Bayer, Germany). A scanning protocol is given in Supplementary Table 1.

### Imaging evaluation

All available MRI examinations were independently evaluated in a randomised order by two experienced board-certified neuroradiologists (TF and FW) who were blinded to clinical information. Each intracranial artery (internal carotid, anterior cerebral, middle cerebral, posterior cerebral, basilar and vertebral arteries) was individually evaluated for signs of inflammation including side (if applicable).

The following location-dependent variables were measured:

- Signal intensity (SI) of vessel wall enhancement on TSE dark blood sequence after contrast.
- SI of vessel wall enhancement on SPACE dark blood sequence after contrast.
- Length of vessel wall enhancement on TSE and SPACE dark blood sequence after contrast (millimetre, mm).
- Circumferential extent of the enhancement of the vessel wall on TSE and SPACE dark blood sequence after contrast (0–360°, 90° increments).
- Extent of the stenosis on 3D-TOF (0–100%, 5% increments).

In contrast to computed tomography (CT) examinations, where Hounsfield units are generally considered comparable, SI measurements on MRI may depend on software and hardware settings. To make the SI measurements taken from the TSE and SPACE dark blood sequences comparable, the “noise”-signal (SI in the air) was taken by Reader 1 (TF). First, the area of pathologic enhancement of the vessel wall was visually identified by visual comparison to the contralateral artery and to unaffected, more distal and proximal segments of the same artery. SI measurements of the vessel wall were taken with our PACS toolbox by placing a region of interest (ROI) in the vessel wall excluding the adjacent vessel lumen and the brain parenchyma. Length of vessel wall enhancement was also measured with our PACS toolbox by measuring the longitudinal extension of the enhancing vessel wall. Extent of circumferential enhancement in SPACE-Sequences was determined by multiplanar reconstruction along the vessel and estimation of the extent of the circumferential enhancement on cross section of the vessel. On TSE, circumferential grading was measured on axial planes by evaluating the anterior and posterior wall at the level of maximal vessel wall enhancement. Second, a plane superior to the vessel lumen was used to evaluate the enhancement of the superior vessel wall, the inferior vessel wall was then evaluated in the same manner. The total extension of circumferential enhancement was calculated by adding enhancement in all four quadrants. In cases where circumferential enhancement differed along the longitudinal course of the vessel, the observed maximum was used. For evaluation of the extent of the stenosis, the 3D TOF Sequence was reconstructed along the affected vessel, measurements of stenosis were conducted in adherence to the North American Symptomatic Carotid Endarterectomy Trial (NASCET) recommendations [18].

In addition, the number of days since the patient started treatment and the patient’s age at the initiation of treatment were recorded.

### Statistical methods

All analyses were conducted in R version 4.0.2, using the ‘pastecs’, ‘psych’, and ‘ggpubr’ packages to calculate and depict descriptive statistics, while generalised multi-level models were fitted with the ‘brms’ and ‘rstan’ packages to allow full Bayesian inference using “Stan”.

#### Interobserver reliability

The extent of agreement between the measurements taken by the two readers was evaluated for each of the five variables, using the intraclass correlation coefficient (ICC) and Cohen’s κ for continuous and categorical variables. Measurements were treated as independent.

#### Statistical models

To investigate the temporal profile of: (i) vessel wall enhancement, (ii) length of vessel wall enhancement, (iii) circumferential extent of enhancement of the vessel wall, and (iv) stenosis, four series of Bayesian generalised multi-level models were generated and examined. Each outcome was expressed as a function of: (i) the number of days since the initiation of treatment, (ii) patient’s age at start of treatment, and (iii) type of blood vessel affected. The relationship between all outcomes and the first predictor variable was clearly non-linear, and a mechanistic model based on a thorough theoretical understanding of the data generative process was lacking. Therefore, natural splines (with internal knots located at the 25th, 50th, and 75th quantile) were incorporated for this predictor. All up to second-order interactions between the different predictor variables were considered, on the precondition that they did not introduce any rank deficiencies in the model matrix. Only interaction terms that led to a more strongly supported model, given the data at hand, were retained and reported below. To account for the multi-level structure of the data, location ID was included as a varying intercept within Patient ID, which in turn was nested within Hospital ID.

Model parameters were estimated by running four independent Markov chains, employing the Hamiltonian Monte Carlo algorithm with NO-U-Turn Sampler, for 8000 iterations each. The first 4000 iterations were used to warm up the algorithm and the last 4000 to sample the joint posterior distribution. Weakly regularising priors were specified to restrict the sampled parameter space, defining normal distributions for fixed intercepts (μ = 0, σ = 10) and effects (μ = 0, σ = 5), while setting Cauchy distributions (x0 = 0, γ = 2) for variance components. Chain mixing, stationarity, and convergence were assessed by visually inspecting trace plots and by insisting on R-hat values of 1.00. This required an increase of the resolution of the sampler by setting the “adapt_delta” argument to 0.999, and allowing a maximal tree depth of 18. Effective sample size of all parameter estimates was > 2500.

To facilitate model convergence and interpretation, all continuous predictor variables were transformed to *z*-scores prior to estimation. Nested models were compared using approximate Leave-One-Out (LOO) cross-validation to identify the most supported model given the data at hand, on the basis of their respective Expected Log pointwise Posterior Density (ELPD LOO; higher values are indicative of more support). Where required, outcome variables were transformed to ensure that all observations fell within the range of possible values given the most appropriate model distribution.

## Results

The search yielded nine cases of PACNS from the Bern University Hospital and 14 from the St. Gallen Cantonal Hospital database giving a total of 23 patients with a total of 221 MRI examinations.

The following 3-T scanners were used: Siemens Magnetom Skyra, Skyra fit, Verio, Vida, Prisma fit and Trio. The following 1.5-T scanners were used: Siemens Magnemtom Area, Avanto, Avanto fit, Symphony and Philipps Intera. Vessel wall imaging was performed on 3-T scanners only.

### Demographical and clinical characteristics of the included patients

Mean age was 50 years (standard deviation ± 16.5 years), range 21–77 years. Fifteen out of 23 (65%) patients were male with a mean age of 52 years (± 16.0 years); mean age of females was 46 years (± 19.0 years), differences were not statistically significant. Median time from first symptom until start of immunosuppressant therapy was 0.4 months, range 0.1–26 months. All patients received a lumbar puncture. DSA was performed in the majority of cases (*n* = 13), in all cases, findings were compatible with PACNS. Brain or meningeal biopsy was performed less frequently (*n* = 6) and results were less clear: in four cases, no signs of vasculitis were seen, in two cases results were unspecific. An overview of the included patients, clinical symptom at presentation and affected vessels is given in Table 1.

**Table 1:** Patient characteristics

Patient ID	Age at start of treatment	Sex	Clinical presentation	kl	Number of locations affected	MRI findings (initial presentation)
BE03	56.6	Male	Dizziness		2 (left MCA, right MCA)	Infarction left posterior MCA territory
BE04	74.5	Male	Dysarthria, right hemiparesis		1 (left MCA)	Infarction left posterior MCA territory
BE05	27.6	Male	Dysarthria, right hemianopsia and hemiparesis		1 (left ICA)	Infarction left posterior MCA territory
BE06	59.3	Female	Headache, dysarthria, nausea		1 (left MCA)	Subarachnoidal haemorrhage left parietal lobe
BE07	62.3	Female	Aphasia, left-sided hemisyndrome		4 (left MCA, left ACA, right MCA, right PCA)	Infarction left ACA and MCA territory
BE08	29.9	Male	Dysarthria		1 (left MCA)	Embolic infarction left MCA territory
BE09	77.5	Male	Left-sided leg weakness		2 (right ACA, left VA)	Embolic infarctions right MCA territory
BE10	22.4	Female	Right-sided hemisyndrome		1 (left ICA)	Left thalamic infarction
BE11	26.1	Male	Left-sided hemianopsia		1 (right PCA)	Right thalamic, right PCA territory infarction
SG01	62.2	Male	Aphasia, right-sided hemiparesis		2 (left ICA, right ICA)	Bilateral MCA territory embolic infarction
SG02	40.8	Female	Right-sided hemiparesis		4 (left MCA, left ICA, right ICA, right ACA)	Infarction left MCA territory
SG03	36.2	Male	Right-sided tingling sensations		3 (right MCA, left MCA, right PCC)	Infarction left MCA territory
SG05	51.5	Male	Dysarthria, right-sided hemisyndrome		3 (left MCA, right MCA, right ACA)	Infarction right ACA territory
SG07	28.8	Female	Left-sided hemisyndrome, reduced vigilance		2 right MCA, right ACA	Infarction right ACA territory
SG08	70.6	Female	Dysarthria, left-sided hemiparesis		3 (left MCA, right ICA, right MCA)	Embolic infarction right MCA territory
SG09	62.4	Male	Left-sided hemiparesis		1 (right ICA)	Infarction right MCA territory
SG10	48.3	Male	Left-sided arm weakness		1 (right MCA)	Infarction right MCA territory
SG11	50.4	Male	Nausea, dizziness, double vision		1 (BA)	Bilateral cerebellar infarctions
SG12	28.0	Female	Right-sided hemianopsia		1 (left PCA)	Infarction left PCA territory and left thalamus
SG14	58.0	Male	Left-sided hemisyndrome		3 (right MCA, left MCA, right ACA)	Infarction right MCA territory
SG15	54.4	Male	Left-sided hemisyndrome		1 (right MCA)	Infarction right MCA territory
SG16	64.2	Female	Numbness right arm		3 (right MCA, left MCA, right PCA)	Infarction right MCA territory
SG17	63.5	Male	Numbness right hand		1 (left MCA)	Embolic infarctions left media territory

*ACA* anterior cerebral artery, *MCA* middle cerebral artery, *PCA* posterior cerebral artery, *VA* vertebral artery, *ICA* internal carotid artery, *BA* basilar artery, *BE* Bern site patient, *SG* St. Gallen site patient

### Description of the data sample

The dataset comprised 402 entries in 43 different localisations in 23 patients from two hospitals and was obtained by evaluating 209 MRI scans. Raw TSE values for vessel wall enhancement were available for 22 patients, whereas raw SPACE values were only available for 15 patients. For 19 localisations across 14 patients, both raw TSE and SPACE values were reported. Measurements of the length of vessel wall enhancement, circumferential extent of enhancement and extent of stenosis were obtained in all 23 patients. Mean time to follow-up MRI was 715 days, standard deviation ± 487: minimum 18 days, maximum 1496 days.

### Interobserver reliability

**Fig. 1:**
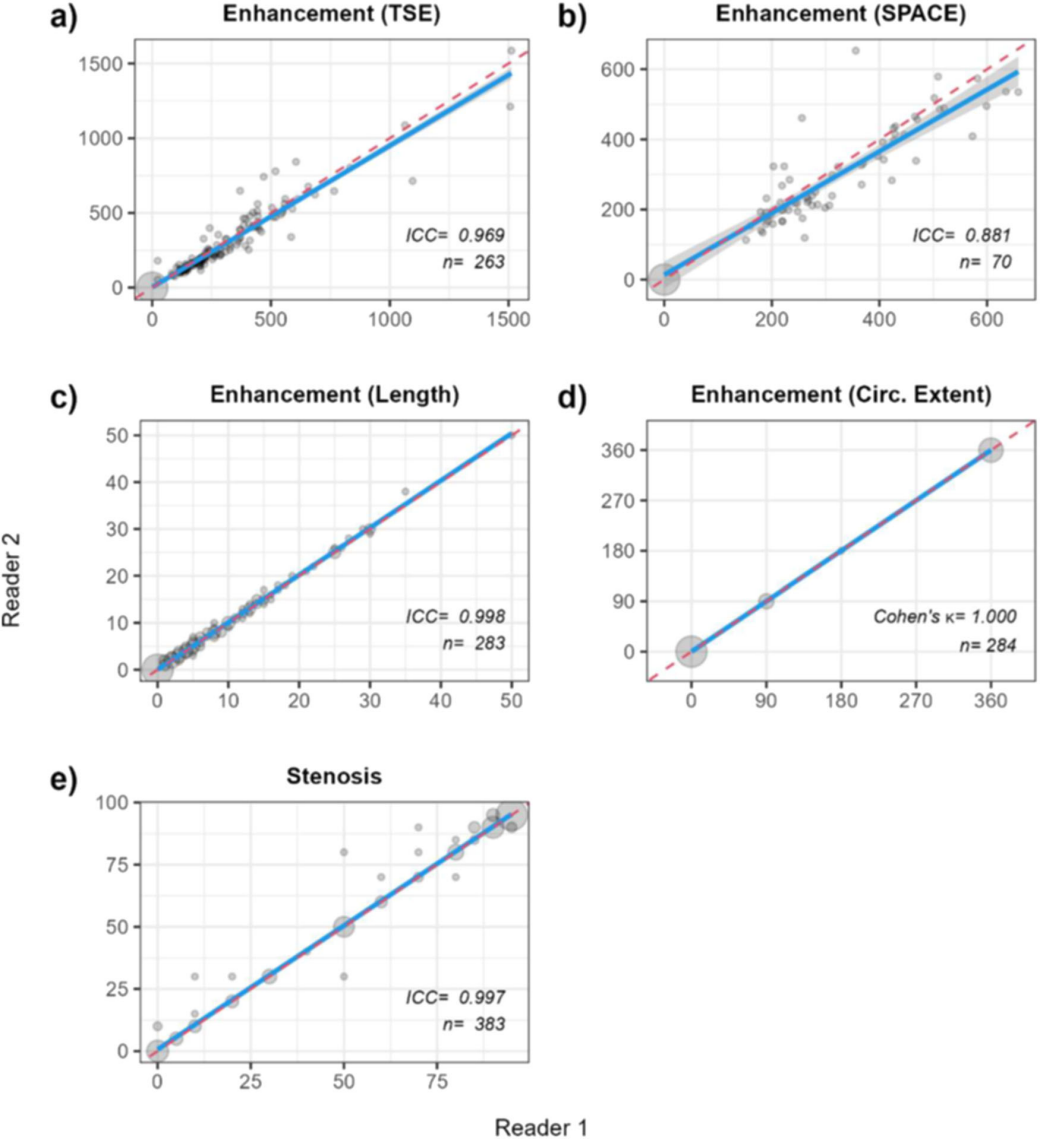
Scatterplots to visually assess the interobserver reliability between the two readers on five key variables measured in 209 MRI images at 43 different locations across 23 patients (**a**–**e**). Blue lines represent the average empirical agreement, along with its associated 95% confidence interval (shaded grey), whereas dotted red lines are lines of perfect agreement (x = y). Note that in panels **a** and **b** the blue line dips below the line of perfect agreement towards the top-right section of both graphs, indicating that towards the high end of the empirical range, Reader 1 scored systematically lower values for these measures than Reader 2 (ICC IntraClass Correlation Coefficient, *n* sample size; diameter of bubbles is proportional to the number of observations)

Test statistics suggest a “good to excellent” agreement. In addition, scatterplots were overlaid with bivariate regression lines (blue) as well as lines of perfect agreement (red; x = y) (Fig. 1). A potential, albeit slight, bias in the scores of the two vessel wall enhancement variables (panels a and b) was observed: Reader 1 tended to consistently report lower values than Reader 2 towards the high end of the observed range.

### Association between TSE and SPACE sequence measurements

The intra-reader association between TSE and SPACE scores (vessel wall enhancement and noise) was obtained from examinations with sequences available (*n* = 19). Observations were treated as independent. ICC values were low: Reader 1: ICC = 0.476, Reader 2: ICC = 0.406, Noise (Reader 1 only): ICC 0.229. This low ICC led to the conclusion that TSE and SPACE values obtained for vessel wall enhancement are not comparable. Therefore, vessel wall enhancement was investigated using standardised TSE metrics exclusively.

### Temporal profile of vessel wall enhancement

**Fig. 2:**
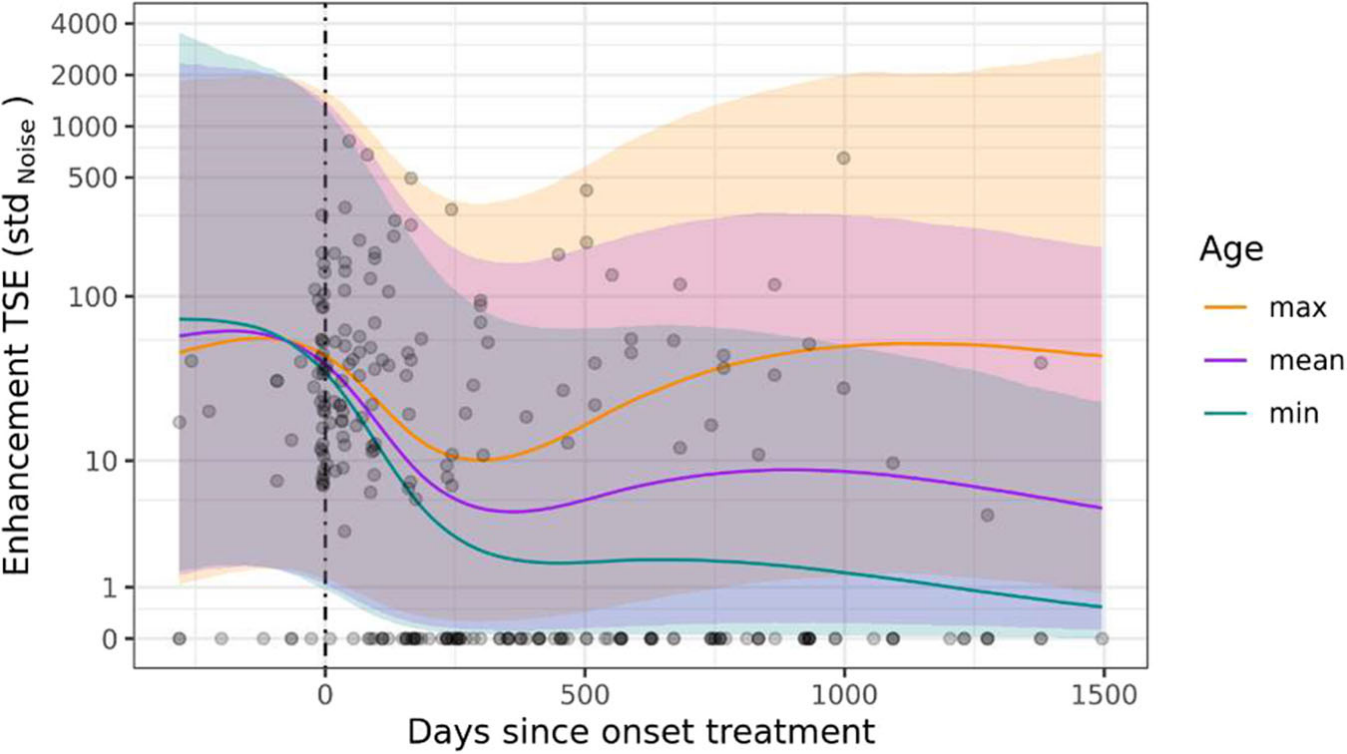
Scatterplot of TSE std. noise values and number of days since the start of treatment, with prediction lines and associated 95% credible intervals from the most supported Bayesian multi-level model. For visualisation purposes, predictions are shown at three discrete values of patient age (minimum = 22.4, mean = 47.0, and maximum = 77.5 years). Note the logarithmic scale on the *y*-axis, as well as the extremely wide credible intervals, indicative of a low signal-to-noise ratio in the data

A visual representation of the standardised TSE metrics for vessel wall enhancement is provided in Supplementary Fig. 1. Given the continuous nature, pronounced right-skew, and hard lower bound of the outcome (i.e., values < 0 are not possible), a lognormal multi-level model was fitted to express Enhancement TSE (std. Noise) as a function of the predictor variables under consideration. To be able to retain 0 scores, and in accordance with statistical convention when dealing with lognormal data, a value of 1 was added to all observations prior to analysis. The amount of support for the series of nested models leading up to the overall preferred model is summarised in Supplementary Table 2. Predictions by this most supported model are superimposed onto the empirical data in Fig. 2.

The most supported model accounts for about half of the total variability in the data (R2 Bayesian = 0.523, or 52.3%), and performs considerably better than a null model consisting of intercepts only (Δ ELPD: mean ± SE = 70.59 ± 11.26), as well as the next most supported model (comprising an Intercept, Days (as a natural spline of *z*-scores) and Age (*z*-scores) as predictor variables; Δ ELPD: mean ± SE = 17.83 ± 7.34). Supplementary Table 2 thus unequivocally shows that vessel wall enhancement as measured by TSE std. Noise scores are best understood in terms of a second-order interaction between a natural spline of the number of days since the start of treatment and patient age, whereas incorporating blood vessel type did not contribute to model performance in a meaningful way. Figure 2 shows how this second-order interaction implies that after the initiation of treatment, blood vessel wall enhancement in all patients initially decreased. Both the rate and extent of this decrease were inversely related to patient age, with younger patients reacting both faster and more strongly to treatment than older patients. Following this initial period of decrease, lasting up to approximately 300 days into treatment, vessel wall enhancement typically remained relatively low and stable in young to average-aged patients, with younger patients possibly exhibiting a plateau at lower TSE std. Noise scores.

A complete disappearance of vessel wall enhancement of at least one vessel after the start of therapy was observed in 11/23 patients. Time intervals are given in Table 2. The earliest observed disappearance was seen 54 days after therapy started. No age-dependency was observed in patients in whom vessel wall enhancement disappeared in at least one vessel (data not shown).

**Table 2:** Time intervals after the start of therapy at which vessel wall enhancement disappeared in at least one artery

	Min.	1st Qu.	Median	3rd Qu.	Max.	*N*
All	54	113	172	244	627	18
A	122	139	156	195.5	235	3
ICA	87	115.2	143.5	171.8	200	2
M	54	172	244	471.5	627	10
P	83	88.5	94	131.5	169	3

Note that BA and VA never reached zero enhancement

*A* anterior cerebral artery, *M* middle cerebral artery, *P* posterior cerebral artery

### Temporal profile of length of vessel wall enhancement

**Fig. 3:**
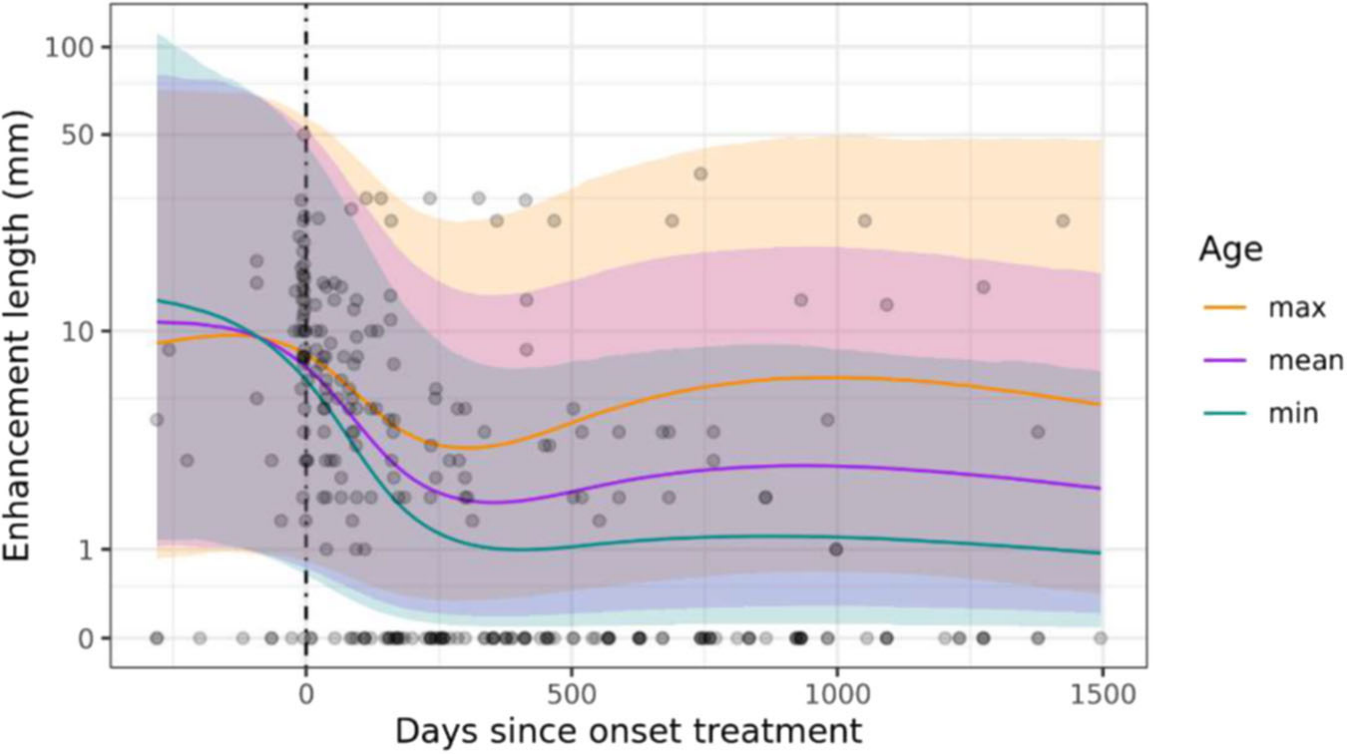
Scatterplot showing the length of vessel wall enhancement and the number of days since the start of treatment with prediction lines and associated 95% credible intervals from the most supported Bayesian multi-level model. For visualisation purposes, predictions are shown at three discrete values of patient age as observed in the sample (minimum = 22.4, mean = 47.0, and maximum = 77.5 years). Note the logarithmic scale of the *y*-axis, as well as the wide credible intervals

A visual representation of the raw temporal profile of the length of vessel wall enhancement is given in Supplementary Fig. 2. As for vessel wall enhancement, a lognormal multi-level model was most suitable to analyse the data, as the outcome exhibited a continuous and right-skewed distribution with a strict lower bound (values < 0 are not possible). To retain 0 observations, a value of 1 was added to all scores prior to analysis. The amount of support for all candidate models nested within the most supported model is summarised in Supplementary Table 3, and predictions made by the preferred model are presented in Fig. 3.

The most supported model explains just shy of two-thirds of the total variability in the data (65.5%), outperforming a null model consisting of intercepts only (Δ ELPD: mean ± SE = 77.36 ± 13.45), as well as the next most supported model (comprising an Intercept, Days (as a natural spline of *z*-scores) and Age (*z*-scores) as predictor variables; Δ ELPD: mean ± SE = 17.64 ± 7.12). Supplementary Table 3 thus implies that the length of vessel wall enhancement is best understood in terms of a second-order interaction between a natural spline of the number of days since the start of treatment and patient age, with no differences between blood vessel types.

Figure 3 exhibits a very similar temporal profile to Fig. 2: following an initial rapid and pronounced decrease, which is inversely proportional to patient age, a period of lower and relatively constant values follows. However, in older patients both the rate and extent of the initial decrease are less pronounced.

### Temporal profile of the circumferential extent of enhancement of the vessel wall

A visual representation of the temporal profile of the circumferential extent of enhancement of the vessel wall is given in Supplementary Fig. 3.

**Fig. 4:**
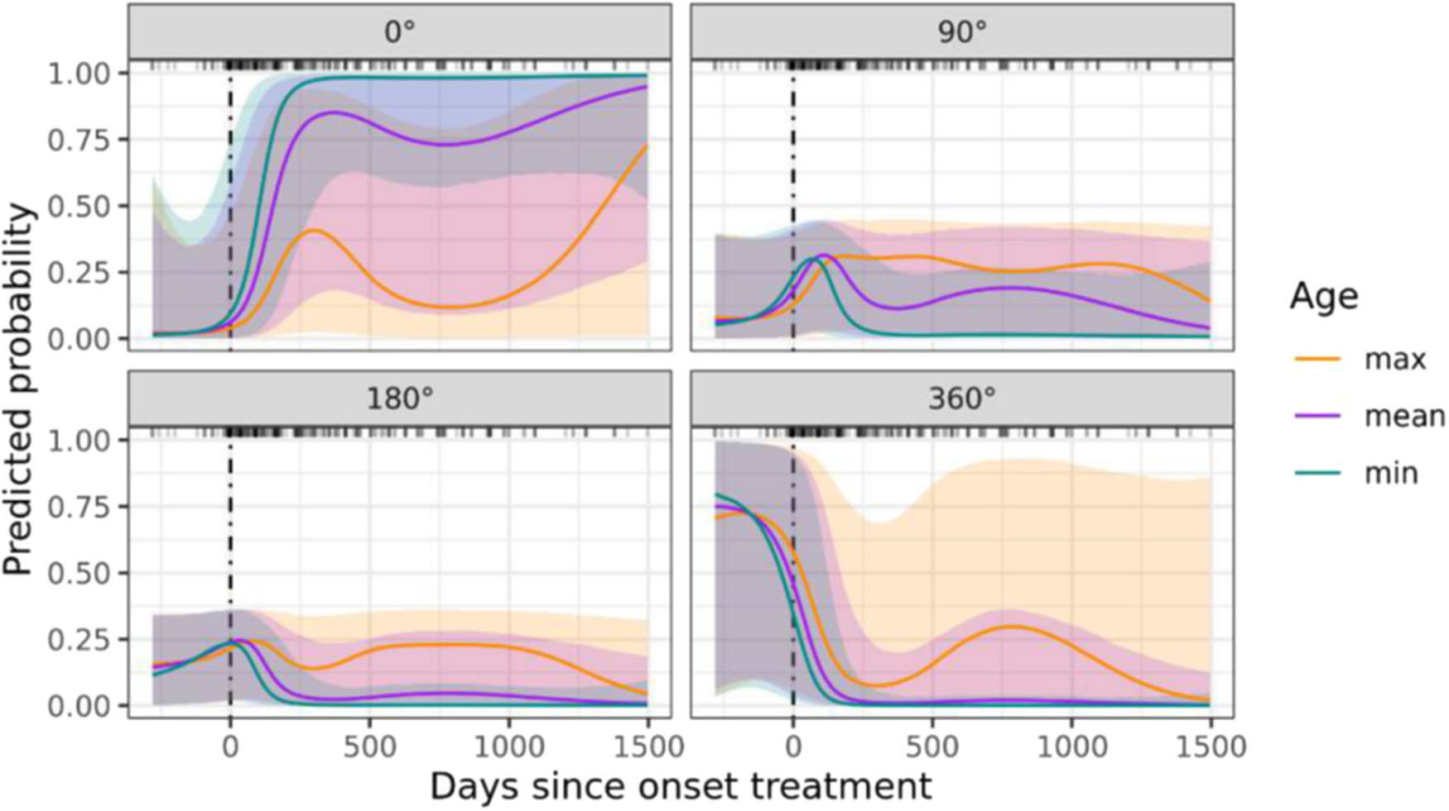
Predicted probabilities for each of the four observed values of circumferential extent of enhancement of the vessel wall as obtained from the most supported Bayesian multi-level model. For visualisation purposes, predictions are shown at three discrete values of patient age that occur in the sample (minimum = 22.4, mean = 47.0, and maximum = 77.5 years). Note the wide credible intervals, especially in the first (circumferential extent = 0°) and fourth (circumferential extent = 360°) panels of the figure

Given the ordinal nature of the data, a cumulative multi-level model with flexible thresholds was fitted. The amount of support generated for all models nested within the model that ultimately gained the most support is shown in Supplementary Table 4. Predicted probabilities for each of the observed outcome categories are given in Fig. 4. The most supported model accounts for a substantial amount of the total variability in the data (65.1%), outperforming a null model consisting of intercepts only (Δ ELPD: mean ± SE = 67.36 ± 11.32). Note that, to calculate an approximate R2-value, samples from the joint posterior distribution were treated as continuous, but this is probably of limited validity (due to the ordinal nature of the outcome). Interestingly, the second most supported model (comprising an Intercept and Days (as a natural spline of *z*-scores) as the sole predictor variable) performed only marginally worse than the most supported model (Δ ELPD: mean ± SE = −5.90 ± 5.20). Nevertheless, Supplementary Table 4 implies that the circumferential extent of enhancement of the vessel wall is best understood in terms of a second-order interaction between a natural spline of the number of days since the start of treatment and patient age, with no differences due to blood vessel type.

The first panel in Fig. 4 shows that the probability of a circumferential extent of 0° increases rapidly, and inversely proportional to patient age, within approximately the first 300 days after initiation of treatment, before reaching relatively constant and age-dependent plateau values. The fourth panel (depicting the predicted probability of a circumferential extent of 360°) shows an approximate mirror image.

### Temporal profile of the extent of stenosis

A visual representation of the temporal profile of the extent of stenosis, expressed in percentages, is given in Supplementary Fig 4. It is noteworthy that for the far majority of localisations, the amount of change over time, if any, is minimal. Based on this observation, a statistical model for the evolution of stenosis was not calculated.

## Discussion

**Fig. 5:**
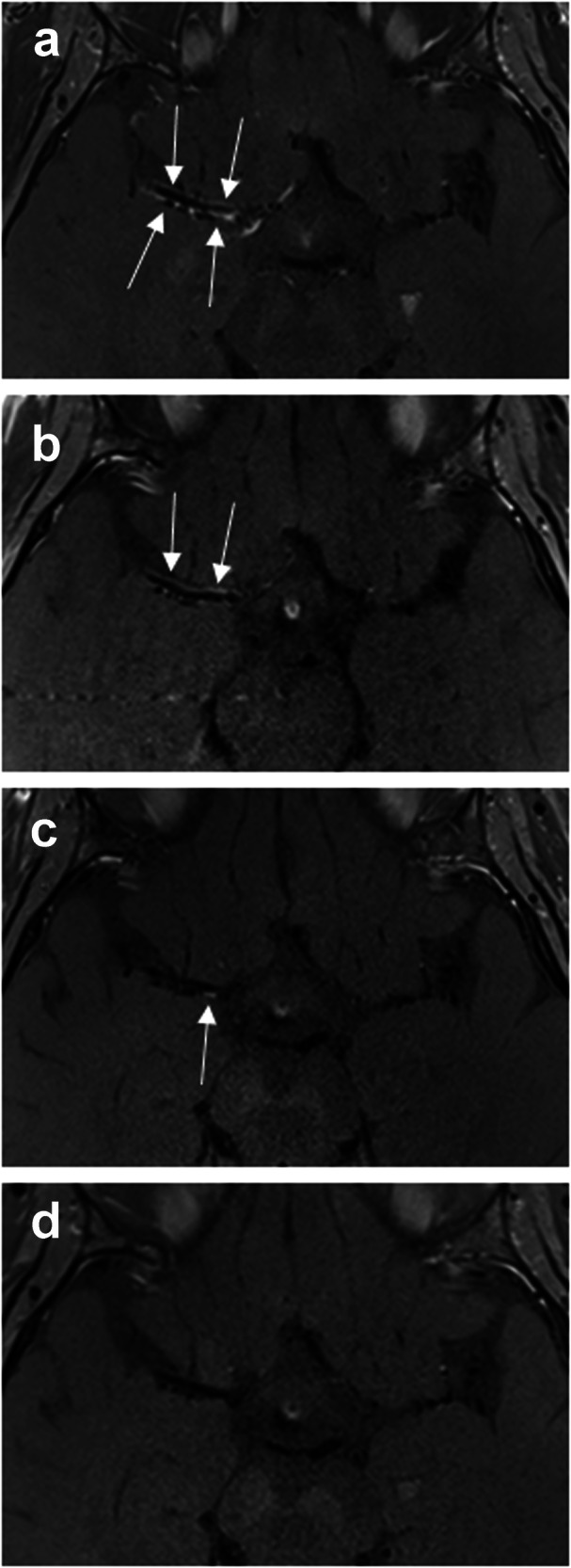
T1-weighted dark blood turbo spin echo MRI with intravenous contrast agent in a 29-year-old female patient with primary angiitis of the central nervous system of the right middle cerebral artery. Vessel wall enhancement (white arrow) decreases over time and finally disappears completely. **a** 13 days before immunosuppression, (**b**) 4 days after treatment start, (**c**) 235 days after treatment start, (**d**) 377 days after treatment start. Note the complete disappearance of the vessel wall enhancement over a long time

This study describes the temporal profile of vessel wall enhancement, length of vessel wall enhancement, circumferential extent of enhancement of the vessel wall, and stenosis as retrospectively reconstructed from MRIs collected from 23 patients with PACNS. Despite noisy data, a rather consistent pattern in the temporal profile of vessel wall enhancement and stenosis emerged across all outcome variables—following immunosuppressant treatment, enhancement of vessel wall, enhancement length, and circumferential extent of enhancement of the vessel wall decreased until approximately 1 year (365 days) after initiation of treatment. Changes were more pronounced in younger patients than in older ones. In approximately half of patients (11/23) a complete disappearance of enhancement in at least one artery was observed after a minimum interval of 54 days after treatment start. An imaging example is given in Fig. 5. Stenosis grading did not show much variation: an initially high stenosis remained high, and a significant reduction of stenosis after initiation of therapy was observed only rarely.

Currently, MRI might be the most important contributor for diagnosing PACNS, since it is abnormal in virtually all affected patients, regardless of biopsy- or angiogram-confirmed diagnosis, which, although very sensitive, has lower specificity [1, 11]. Imaging differentials include atherosclerotic plaque, which may exhibit similar clinical symptoms regardless of the severity of the stenosis when intraplaque haemorrhage and inflammation occurs [19], reversible cerebral vasoconstriction symptom (RCVS) [20], or moya-moya disease [21].

Few studies have described imaging changes in patients with PACNS over time. One recent study assessed 45 patients and 120 vessels with a median follow-up time of 239.5 days. Progressive enhancement was reported in 10/120 cases (8.3%), a stable situation in 52/120 cases (43.3%) and regression or no enhancement in 58/120 cases (48.3%). Per patient and MRI scan, vessel wall enhancement was rated as progressive in 5/55 cases (9.1%), as stable in 25/55 cases (45.5%) and as regressive or no enhancement in 25/55 cases (45.5%). Complete resolution of vessel wall enhancement was seen, especially after mid-term follow-up (3–12 months), in 31.8% of available vessels and at long-term follow-up (> 12 months) in 38.1% of locations assessed [17]. This data supports the findings of our study, where a decrease in vessel wall enhancement was observed until approximately 1 year after treatment started with approximately half of vessels reaching zero enhancement.

## Limitations

The major limitations of this study were its retrospective nature and the nonhomogeneous MRI datasets. MRI scans were performed when clinically indicated and the total number of scans and scan intervals differed between patients. Short-term cortisol (three days) therapies were not systematically evaluated, since cortisol was sometimes given more than once before the start of the immunosuppressant treatment and was sometimes omitted. Timeframes for short-term cortisol therapy were largely heterogeneous. From the data readout, it appears that this short-term cortisol therapy does not affect vessel wall enhancement. Although a standard treatment regimen exists, which the majority of the PACNS patients undertook, not all patients received the same medication for the same time periods. Adaptations were made as clinically indicated. Comparability of TSE and SPACE dark blood sequences was deemed insufficient and therefore the sample size for evaluation of vessel wall enhancement had to be reduced. Furthermore, some minor limitations are related to the statistical model: the relatively small number of observations per vessel and the unbalanced data, which make model predictions associated with a large degree of uncertainty.

## Conclusion

This study systematically analysed the time course of vessel wall enhancement in patients with PACNS undergoing treatment with immunosuppressants. Our general conclusions can serve as a reference for radiologists and clinicians on what to expect on follow-up MRI of their patients with PACNS.

### Supplementary information

ELECTRONIC SUPPLEMENTARY MATERIAL

## Data Availability

The dataset analysed during this study are available from the corresponding author on reasonable request.

## Abbreviations

ICC: Intraclass correlation coefficient
LOO: Leave one out
PACNS: Primary angiitis of the central nervous system
SI: Signal intensity
SPACE: Sampling perfection with application optimised contrast using different flip angle evolution
TOF: Time of flight
TSE: Turbo-spin echo
