# Supplementary material for: Temporal evolution of primary angiitis of the central nervous system (PACNS) on MRI following immunosuppressant treatment
Source: Insights Imaging. 2024 Jun 9;15:140. doi: 10.1186/s13244-024-01710-y (PMC11162979; doi:10.1186/s13244-024-01710-y)
Supplement: Supplementary file 1 — ELECTRONIC SUPPLEMENTARY MATERIAL [file 13244_2024_1710_MOESM1_ESM.pdf]

Temporal evolution of primary angiitis of the central nervous system (PACNS) on MRI following immunosuppressant treatment

ELECTRONIC SUPPLEMENTARY MATERIAL

Supplementary Table 1 PACNS MRI 3T (Siemens Magnetom Skyra) Scanning protocol.

Sequence	Orientation	Slice Thickness	Time of acquisition
Localizer	Auto Align	1.4mm	00:39min
T2 turbo spin echo	transversal	4mm	01:33min
T1 SPACE dark blood (pre- / post contrast)	sagittal	3.0mm	03:26min
Time of flight flow 3D	transversal	0.5mm	05:18min
Diffusion resolve B0/B1000	transversal	4.0mm	02:45min
T1 mprage iso	transversal	0.85mm	04:46min
T2 SPACE dark fluid	sagittal	1.0mm	02:17min
T2 Susceptibility weighted imaging	transversal	1.8mm	04:08min
T1 spin echo blood suppressed* (pre- / post contrast)	transversal	2.0mm	04:43min

*T1 spin echo blood suppressed is acquired upon radiologist's request after review of the T1 SPACE dark blood sequence. Reasons are for example: 1. unclear signal at the level of the stenosis 2. only T1 spin echo blood suppressed sequence and no SPCACE in the prior examination.

Supplementary Table 2 Vessel wall enhancement

	$ELPD_{LOO}$	SE
Intercept	-1002.30	35.34
Days (natural spline of z-scores)	-949.59	36.64
Age (z-scores)	-949.55	36.77
Blood vessel	-949.91	36.81
Days (natural spline of z-scores): Age (z-scores)	-931.72	36.46

Comparison (analogous to a Type I Analysis of Deviance under a Maximum Likelihood paradigm) of increasingly complex models to quantify vessel wall enhancement (as measured by std. noise scores), leading up to the model that gained most support given the data at hand. Expected Log pointwise Posterior Densities (ELPD) were estimated through Leave-One-Out (LOO) cross-validation.

Supplementary Table 3 Statistical model for length of vessel wall enhancement

	ELPD $_{LOO}$	SE
Intercept	-709.98	26.36
Days (natural spline of z-scores)	-650.49	26.49
Age (z-scores)	-650.25	26.73
Blood vessel	-650.56	26.78
Days (natural spline of z-scores): Age (z-scores)	-632.61	26.37

Comparison (analogous to a Type I Analysis of Deviance under a Maximum Likelihood paradigm) of increasingly complex nested models to quantify the length of vessel wall enhancement (in mm), leading up to the model that gained most support given the data at hand. Expected Log pointwise Posterior Densities (ELPD) were estimated through Leave-One-Out (LOO) cross-validation.

Supplementary Table 4 Statistical model for circumferential extent of vessel wall enhancement

	ELPD $_{LOO}$	SE
Intercepts	-325.67	12.96
Days (natural spline of z-scores)	-263.67	14.45
Age (z-scores)	-263.87	14.50
Blood vessel	-265.16	14.92
Days (natural spline of z-scores): Age (z-scores)	-257.77	14.65

Comparison (analogous to a Type I Analysis of Deviance under a Maximum Likelihood paradigm) of increasingly complex models to predict the circumferential extent of vessel wall enhancement (measured in five ordinal categories, of which only four were observed), leading up to the model that gained most support given the data at hand. Expected Log pointwise Posterior Densities (ELPD) were estimated through Leave-One-Out (LOO) cross-validation.

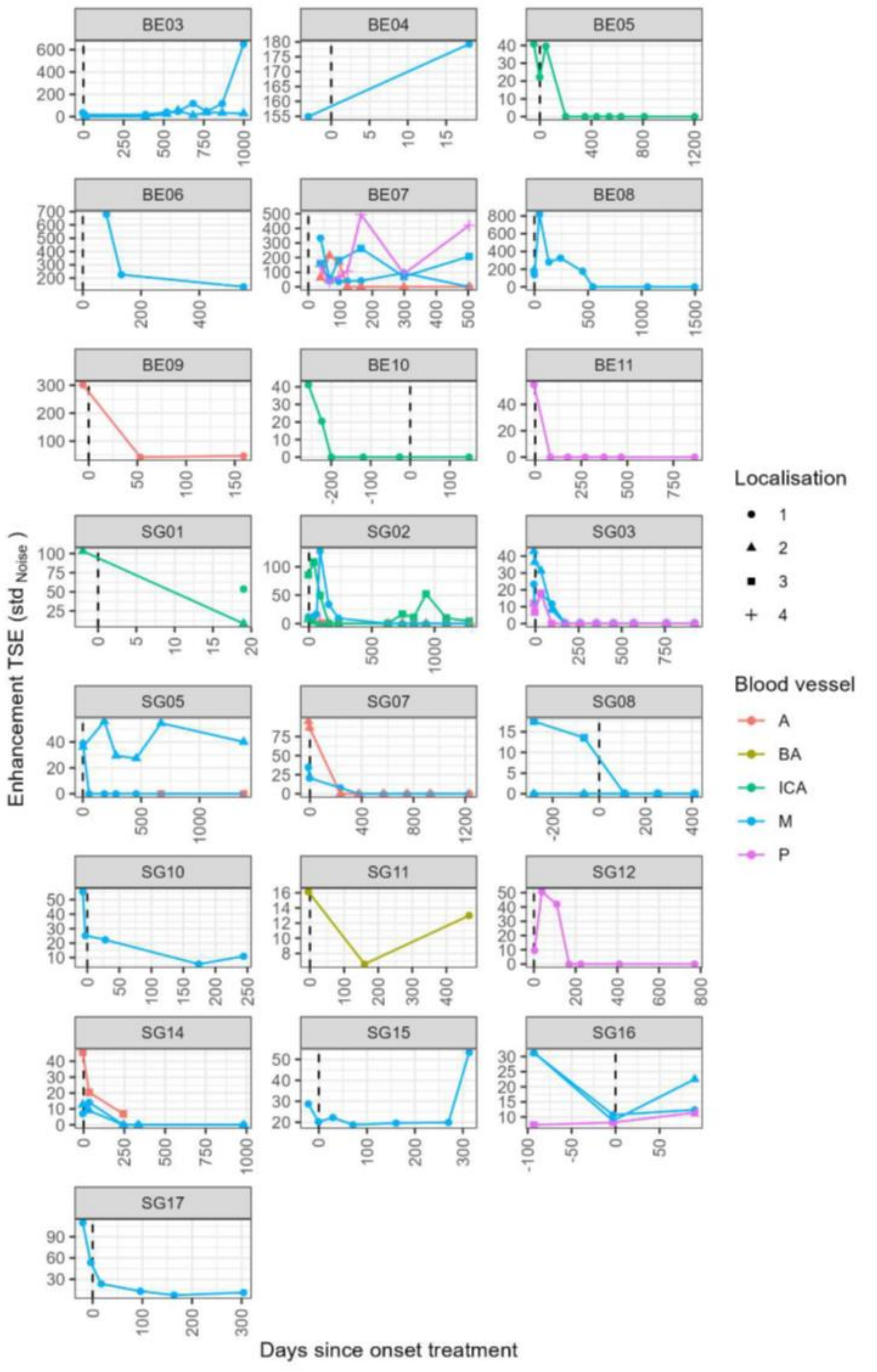

Supplementary Figure 1: Grouped scatter plots for visualizing the temporal pattern in vessel wall enhancement as quantified by turbo spin echo TSE std. Noise scores (n observations = 259, n localisations = 41, n patients = 22, n hospitals = 2). The dashed vertical line indicates the time at which immunosuppressive treatment commenced. A = anterior cerebral artery, BA = basilar artery, ICA = internal carotid artery, M = middle cerebral artery, P = posterior cerebral artery, BE = Bern site patient, SG = St. Gallen site patient.

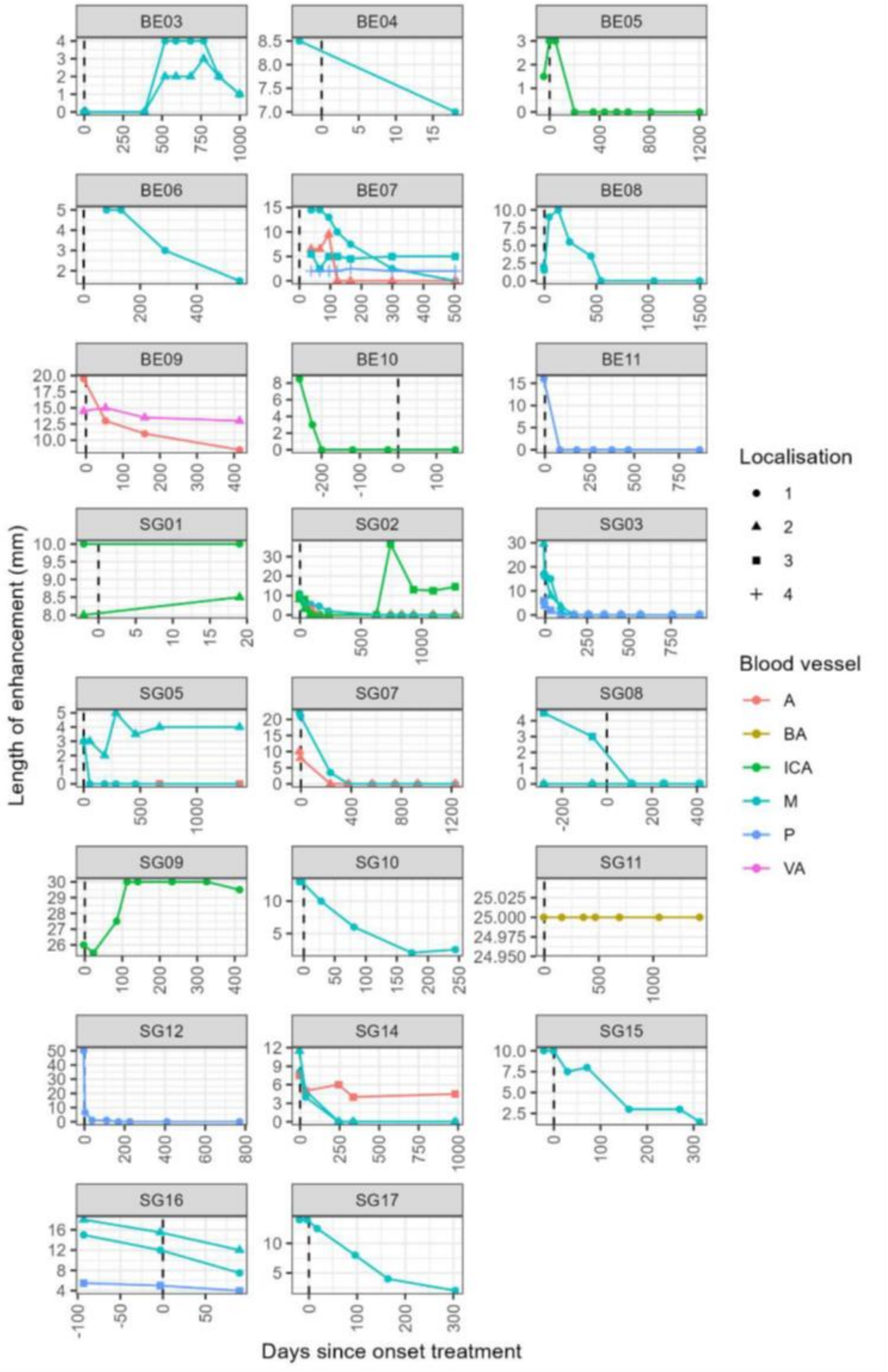

Supplementary Figure 2 Grouped scatter plots for visualising the temporal pattern in the length of vessel wall enhancement (n observations = 283, n localisations = 43, n patients = 23, n hospitals = 2). The dashed vertical line indicates the time at which immunosuppressive treatment commenced. A = anterior cerebral artery, BA = basilar artery, ICA = internal carotid artery, M = middle cerebral artery, P = posterior cerebral artery, BE = Bern site patient, SG = St. Gallen site patient.

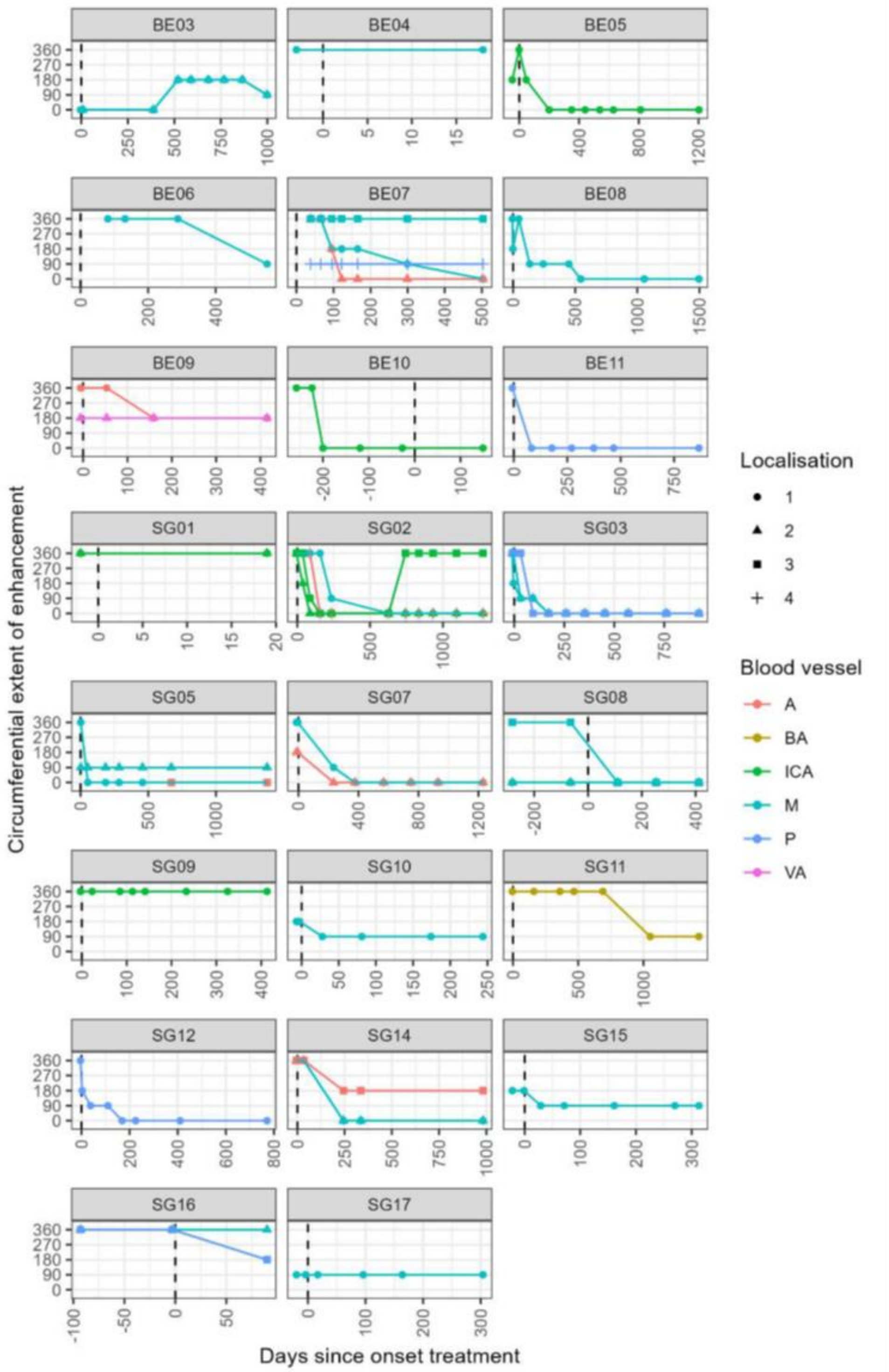

Supplementary Figure 3 Grouped scatter plots for visualising the temporal pattern in the circumferential extent of vessel wall enhancement (n observations = 284, n localisations = 43, n patients = 23, n hospitals = 2). The dashed vertical line indicates the time at which immunosuppressive treatment commenced. A = anterior cerebral artery, BA = basilar artery, ICA = internal carotid artery, M = middle cerebral artery, P = posterior cerebral artery, BE = Bern site patient, SG = St. Gallen site patient.

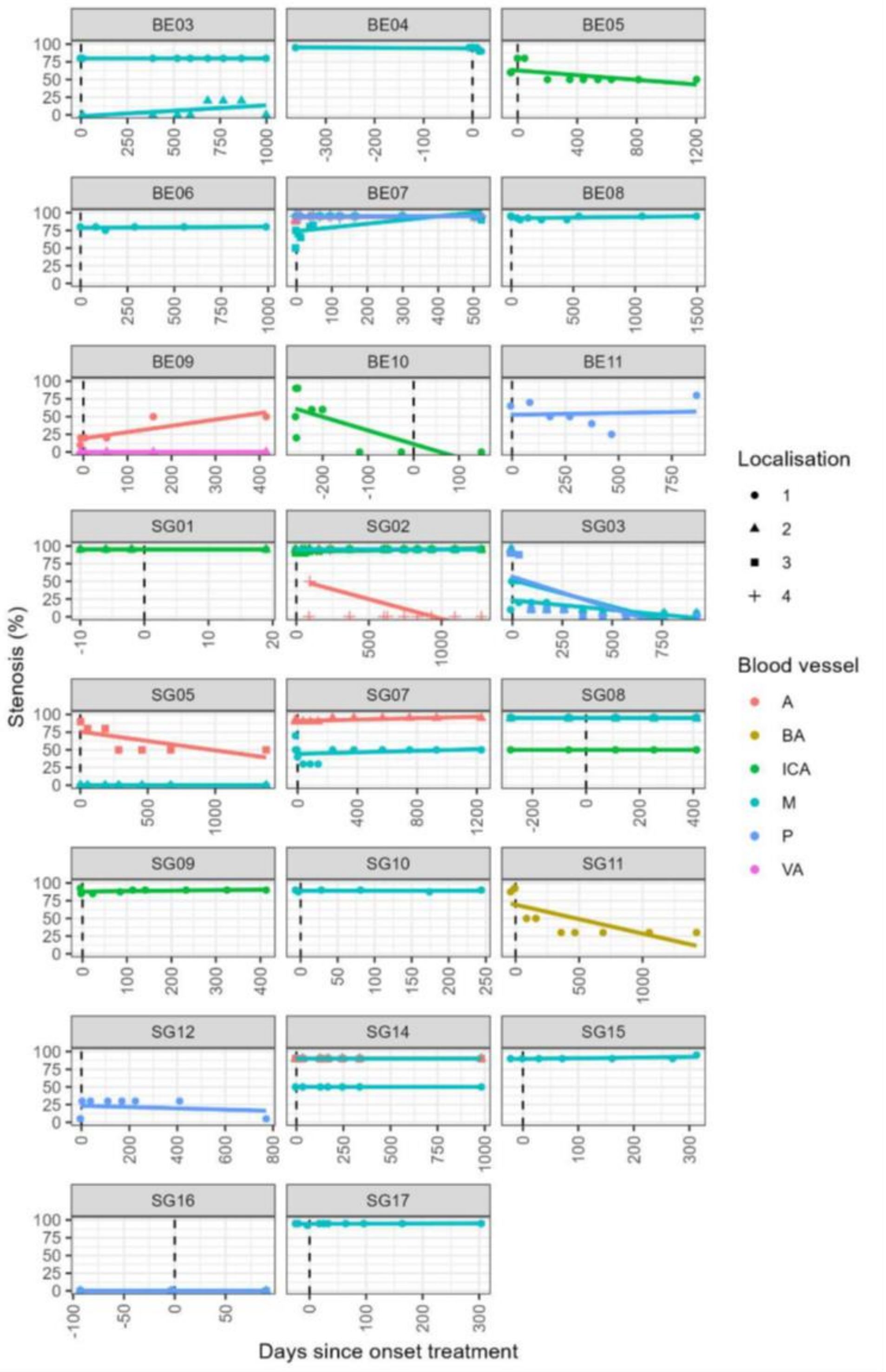

Supplementary Figure 4 Grouped scatterplots for visualizing the temporal pattern in stenosis (n observations = 383, n hospitals =2, n localizations = 43, n patients = 23, n hospitals = 2). The dashed vertical line indicates the time at which immunosuppressive treatment commenced. BE = Bern site patient, SG = St. Gallen site patient.
